# Mind over matter: mindfulness as a buffer against workplace incivility

**DOI:** 10.3389/fpsyg.2024.1409326

**Published:** 2024-08-14

**Authors:** Gonchakhanim Huseynova, Mehmet İslamoğlu

**Affiliations:** Faculty of Business and Economics, Eastern Mediterranean University, Gazimagusa, Türkiye

**Keywords:** co-worker incivility, emotional exhaustion, turnover intention, mindfulness, healthcare organizations, Job Demand-Resources theory

## Abstract

This study examines the relationship between co-worker incivility, emotional exhaustion, mindfulness, and turnover intention among nurses in public and private hospitals in North Cyprus. Drawing upon the Job Demand-Resources theory, the research aims to contribute to the existing literature by investigating the mediating role of emotional exhaustion and the moderating effect of mindfulness on the relationship between co-worker incivility and turnover intention. Data were collected from 238 nurses through questionnaires, and structural equation modeling was employed for data analysis. The results indicate a positive association between co-worker incivility and emotional exhaustion, as well as between co-worker incivility and turnover intention. Emotional exhaustion was found to mediate the relationship between co-worker incivility and turnover intention. Furthermore, mindfulness was identified as a moderator, attenuating the negative impact of co-worker incivility on turnover intention. The findings underscore the importance of addressing workplace incivility and promoting mindfulness to mitigate turnover intentions among nurses. Practical implications include the implementation of interventions to foster a supportive work environment and enhance nurses' emotional wellbeing.

## 1 Introduction

Workplace incivility, a growing area of concern, has been extensively studied due to its significant negative impact on employee performance and overall well-being. Numerous studies, including those by Cho et al. (2016), Hur et al. (2016), Kavaklı and Yildirim (2022), Moon and Morais (2022), and Rahim and Cosby (2016), have demonstrated a strong connection between coworker incivility and employee turnover intention. This study explores the complex relationships between coworker incivility, turnover intention, emotional exhaustion, and mindfulness, particularly within the nursing profession in North Cyprus.

The chosen variables—coworker incivility, turnover intention, emotional exhaustion, and mindfulness—are critical for several reasons. Coworker incivility refers to low-intensity deviant behavior with ambiguous intent to harm the target, violating workplace norms for mutual respect (Agarwal et al., 2023). The healthcare sector, particularly nursing, is characterized by high-stress environments where coworker interactions significantly impact employee wellbeing and job satisfaction. Nurses often experience high levels of emotional exhaustion due to the demanding nature of their work and frequent exposure to stressors, including incivility from coworkers.

Emotional exhaustion is defined as a state of feeling emotionally drained and depleted of emotional resources (Bayighomog et al., 2023). It is a key component of burnout and a strong predictor of turnover intention, which is the likelihood that an employee will leave their job voluntarily. Turnover intention is a significant concern in nursing, where high turnover rates can compromise patient care and increase organizational costs. Mindfulness, defined as the state of being fully present and engaged in the moment without judgment (Calin and Ginara, 2023), has emerged as a potential buffer against the negative effects of workplace stressors. It enhances emotional regulation and resilience, thereby reducing the impact of incivility and emotional exhaustion on turnover intention. This study investigates these variables within the nursing profession in North Cyprus, to address specific gaps in the literature regarding the impact of workplace incivility in this regional and professional context.

This research is grounded in the Job Demands-Resources (JD-R) model, which posits that job demands (e.g., coworker incivility) can lead to strain (e.g., emotional exhaustion) unless mitigated by job resources (e.g., mindfulness) (Huang et al., 2022). By examining these relationships, this study aims to contribute to a deeper understanding of how workplace incivility influences turnover intention and how mindfulness can serve as an effective intervention.

The study utilizes a quantitative research design, surveying nurses in public and private healthcare settings in North Cyprus. Data will be collected through standardized questionnaires measuring coworker incivility, emotional exhaustion, mindfulness, and turnover intention. The analysis employed is structural equation modeling (SEM) to test the hypothesized relationships among the variables, providing robust statistical evidence of the direct and indirect effects within the proposed framework. The primary aim of this study is to examine the complex interplay between coworker incivility, emotional exhaustion, turnover intention, and mindfulness within the nursing profession in North Cyprus. This study aims to provide valuable insights and practical recommendations for improving workplace environments, enhancing employee wellbeing, and reducing turnover rates in the healthcare sector by addressing these issues.

The present study highlights the imperative nature of addressing work-related stress and its associated factors that contribute to emotional exhaustion and intentions to quit among employees. Moreover, the contextual background of North Cyprus adds an additional layer of significance to the present research. The purpose of this study is to examine the potential impact of various cultural, social, and organizational factors on the occurrence and consequences of coworker incivility in North Cyprus. Understanding the contextual factors that may contribute to the prevalence and repercussions of such uncivil behavior is crucial for developing effective strategies to mitigate its negative effects. Cultural factors play a significant role in shaping interpersonal dynamics within a given society. In the case of North Cyprus, the unique cultural characteristics of the region may influence the occurrence of coworker incivility. According to Adeshola et al. (2023), North Cyprus is evolving into a multicultural society, so organizations must adapt their cultural values to prevent potential issues in the future, which is expedient to research the country of context.

Additionally, cultural norms and values regarding appropriate workplace behavior, communication styles, and power dynamics can either foster or discourage uncivil interactions among coworkers. The present study was conducted within a specific context, thereby providing valuable insights into the unique challenges encountered by nurses in North Cyprus or other developing countries. Furthermore, the findings of this study will contribute to the limited body of research on workplace incivility in the region. Moreover, the inclusion of respondents who are nurses enhances the significance and practicality of the study. The significance of nurses as a vital workforce within the healthcare industry cannot be overstated, as their turnover has the potential to yield profound implications for both patient care and organizational achievements.

This study contributes to the advancement of knowledge in several ways. Firstly, it fills a gap in the literature by focusing on the under-researched context of North Cyprus, providing insights into the specific challenges faced by nurses in this region. The cultural, social, and organizational characteristics of North Cyprus can be a factor that determines the extent and impact of coworker incivility. Secondly, it highlights the importance of mindfulness as a potential mitigating factor, offering practical implications for organizational policies and training programs to reduce turnover intentions. Finally, by integrating coworker incivility, emotional exhaustion, mindfulness, and turnover intention into a single framework, which is different from past studies related to this subject matter, this study provides a comprehensive understanding of these interrelated phenomena, thereby advancing theoretical and empirical knowledge.

The subsequent sections include the theoretical framework and the development of hypotheses, which are subsequently followed by the methodology employed, results of analysis, discussion of results, and conclusion.

## 2 Literature review

### 2.1 Definition of variables

Co-worker incivility is characterized by low-intensity deviant behavior with ambiguous intent to harm the target, which violates workplace norms of mutual respect. Such behaviors are generally rude and discourteous, reflecting a lack of regard for others (Lin et al., 2023). Incivility among co-workers can manifest in various ways, such as ignoring, excluding, or being condescending toward colleagues. The prevalence of incivility in the workplace has been shown to contribute to a toxic work environment, leading to negative outcomes for both individuals and organizations, including decreased job satisfaction, increased stress, and higher turnover rates (Kavaklı and Yildirim, 2022). In healthcare settings, incivility can occur among nurses, physicians, and other healthcare professionals, significantly affecting teamwork and patient care. Studies have shown that incivility among healthcare workers can lead to increased stress, reduced job satisfaction, and compromised patient safety (Madden and McAlister, 2022). The high-pressure environment and hierarchical structures in healthcare settings often exacerbate these behaviors, making it a critical issue to address for improving workplace dynamics and patient outcomes.

Emotional exhaustion refers to the state of feeling emotionally overextended and depleted of one's emotional resources, often as a result of prolonged exposure to stressors at work. It is a central component of burnout and is characterized by feelings of fatigue, frustration, and an inability to cope effectively with work demands (Belji Kangarlou et al., 2022). Emotional exhaustion can lead to various negative consequences, including reduced job performance, lower job satisfaction, and increased absenteeism (Parray et al., 2023). The relationship between workplace stressors, such as co-worker incivility, and emotional exhaustion is well-documented, highlighting the significant impact that a negative work environment can have on employees' psychological wellbeing (Wang et al., 2023). The intense nature of healthcare work, involving long hours, high patient loads, and frequent exposure to suffering and death, contributes to emotional exhaustion. This condition can lead to decreased job performance, impaired decision-making, and increased absenteeism, ultimately affecting patient care quality (Siddique et al., 2024).

Turnover intention is the conscious and deliberate willingness to leave the organization or job position. It is a critical antecedent to actual turnover behavior (Lu et al., 2017) and is influenced by multiple factors, including job satisfaction, organizational commitment, and work-related stress (Yang et al., 2017). High turnover intention is often indicative of underlying issues within the workplace, such as poor management practices, lack of career advancement opportunities, and interpersonal conflicts. Turnover intention has significant implications for organizations, as high turnover rates can lead to increased recruitment and training costs, loss of organizational knowledge, and disruption of team dynamics (Xu et al., 2022). High turnover intention among healthcare staff can lead to staff shortages, increased workload for remaining employees, and disruptions in patient care (Xu et al., 2023). Factors contributing to turnover intention in healthcare include poor management practices, lack of career advancement opportunities, and negative work environments characterized by incivility and stress (Lin and Li, 2023). Addressing these issues is crucial for retaining skilled healthcare professionals and ensuring continuous, high-quality patient care.

Mindfulness is defined as the mental state achieved by focusing one's awareness on the present moment, while calmly acknowledging and accepting one's feelings, thoughts, and bodily sensations (Kaya et al., 2024). Rooted in ancient contemplative practices, mindfulness has been increasingly incorporated into modern psychological and organizational practices due to its benefits in enhancing mental clarity, emotional regulation, and overall wellbeing (Gonaduwage et al., 2024). In the workplace context, mindfulness has been shown to mitigate the adverse effects of stress, improve employee wellbeing, and foster a more positive work environment (Dhaka et al., 2024). By promoting a present-focused and non-judgmental awareness, mindfulness can help individuals better manage the emotional challenges associated with workplace stressors, such as incivility, thereby reducing emotional exhaustion and turnover intention (Lin et al., 2022). Mindfulness practices have been integrated into healthcare settings to help professionals manage stress, improve emotional regulation, and enhance wellbeing (Seidel et al., 2021). Additionally, in healthcare, mindfulness has been shown to reduce burnout, improve patient interactions, and enhance overall job satisfaction (Sauvain-Sabé et al., 2023). By fostering a present-focused and non-judgmental awareness, mindfulness can help healthcare professionals cope with the emotional challenges and high demands of their work, thereby reducing the negative impacts of stress and incivility (Chmielewski et al., 2021).

### 2.2 Theoretical framework

**Figure 1:**
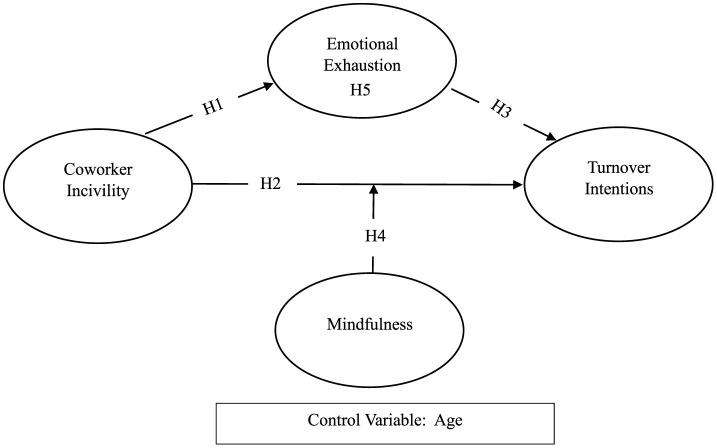
Research model.

Schaufeli and Bakker (2004) JD-R (Job Demand-Resources) model builds up a theoretical foundation for the relationships between coworker incivility, employee turnover, the role of mindfulness, and emotional exhaustion which is demonstrated in Figure 1. According to the JD-R model, job demands are defined as those aspects of work that require continuous physical, psychological, social, or organizational efforts. This is not without cost, as it may lead to the exhaustion of emotional resources. Namin et al. (2021) state that coworker incivility should be recognized as one of the work-related demands. This entails that social aspects of the workplace that incur much management effort and hence have negative emotional implications for employees should be seen as job demands. Emotional exhaustion as a stress factor relates to the fact that employees with such kind of problems are less likely to leave the organization, according to studies. On the other hand, the personnel who are not emotionally exhausted show a positive attitude toward their work, have good performance, and therefore stay in organizations longer.

While on the one hand mindfulness is a great tool for reducing the role of coworker incivility and emotional exhaustion, it can be seen as a job resource on the other hand (Hyland et al., 2015). Mindfulness has been proven to be related to multiple benefits such as lowering stress levels and increasing the feeling of wellbeing (Hyland et al., 2015). Staff members engaging with mindfulness may be skilled in managing emotions while responding more courteously when dealing with coworker rudeness; this will decrease emotional exhaustion, resulting in lower intention to leave.

### 2.3 Hypotheses development

#### 2.3.1 Coworker incivility and emotional exhaustion

During the process of interaction between coworkers, it is possible to experience an emotional drain which may result in anger and frustration (Lee and Madera, 2021). Through time, the person who has been exposed for too long may end up draining his or her emotional resources until their emotional reserves are all used up and experiences emotional exhaustion. Furthermore, workplace incivility among colleagues frequently leads to ongoing psychological stress. When employees are constantly exposed to workplace incivility from their colleagues, they get chronically stressed and anxious, causing a depletion of their psychological resources (Woo and Kim, 2020). With the increasing presence of negativity, individuals find it hard to cope and the stress accumulates to emotional exhaustion. Xia et al. (2022) asserted that coworkers' uncivil behavior usually ends up in interpersonal conflicts and damaged relationships. In this way, the conflicts may be emotionally draining because they consume much of the emotional energy of the individuals who are trying to deal with these difficult situations (Unufe and Igieseri, 2021). Along with the emotional impact of hostile exchanges, these disputes may intensify the emotional exhaustion that the individuals involved are experiencing (Huang, 2023). Furthermore, coworker incivility can cause cognitive rumination (Su et al., 2022), which is the process of frequently thinking about negative experiences and insulting behavior that individuals have been exposed to. With individuals continuously recalling and emotionally reacting to incivility events, this process of rumination may lead to heightened emotional distress and exhaustion.

Research consistently finds that the civility of coworkers is directly related to wellbeing (Gordon et al., 2023). Emotional exhaustion is a usual sign of reduced wellbeing, as people are subjected to emotional wear and tear because of prolonged incivility, which leads to a decrease in their overall emotional and mental health (Abas et al., 2020). The JD-R Model offers a theoretical basis for the link between coworker incivility and feelings of emotional exhaustion. In this context, workplace rudeness is a source of job demand that, when excessive, can lead to emotional exhaustion and burnout. The daily exposure to unkind unfriendliness can be compared to the stress of a demanding job that gradually wears out the emotional reserves of an individual. According to Karatepe et al. (2019), coworker incivility is extremely powerful in increasing emotional exhaustion. The study also mentions the moderating effect of perceived organizational support and emotional regulation capacity in reducing the extent to which coworker incivility influences emotional exhaustion. As Naeem et al. (2020) reported, employees might become negatively affected by their coworker's rude or insolent behavior. Therefore, emotional exhaustion is associated with workplace incivility. Moreover, Hur et al. (2014) introduced a serial multiple mediator model, which indicated that both coworker and customer incivility are associated with emotional exhaustion in the service industry. This result strengthens the evidence that there is a connection between coworker incivility and emotional exhaustion. Therefore, we posit that:

*Hypothesis 1: The relationship between coworker incivility and emotional exhaustion is positive*.

#### 2.3.2 Coworker incivility and turnover intention

Workers might develop psychological stress due to coworker incivility which consists of inconsiderate behavior (Kim and Qu, 2019). Such actions lead to the establishment of an atmosphere where the employees feel distressed, anxious, and filled with negative feelings. Employees who are experiencing psychological distress may try to find an escape from the reason for distress, which is usually expressed through an intention to leave the organization. Another strong predictor of job dissatisfaction is verbal aggressiveness from coworkers (Moon and Morais, 2022). It is the specific case of someone being personally insulted, berated, or, otherwise, the aggression caused by the colleague's verbal hostility. Consequently, those who may experience consistent incivility from their coworkers are more likely to be dissatisfied with their jobs. This discontent might reduce their desire to continue in their current role which in turn, may breed in them the appetite for looking for another job.

While the uncivil behavior of coworkers can be a very strong factor that decreases the organization's engagement (Wang and Chen, 2020). Employees can feel detached and lonely when they see disrespectful or unprofessional conduct among other employees. A decrease in commitment may increase the probability of an employee leaving an organization, and work incivility may impact an employee's job performance negatively (Gordon et al., 2023). The emotional cost of incivility may prevent an employee from contributing effectively to the teamwork, focusing on work, or performing competently at their job. The decline in employee performance may make them feel less indispensable within an organization and increase the chance of them seeking alternative employment where they might expect better treatment.

Workplace rudeness can be categorized under job demand in the JD-R Model, and in extreme cases, it can result in employee burnout including emotional fatigue. A person who is under such stress usually plans to leave the job. This viewpoint underlines the position of emotional exhaustion as a moderator between interpersonal incivility and turnover intention, implying that it has negative consequences on employees' psychological health and the decision to leave an organization. Moreover, job satisfaction can play a role in the process of working with incivility and leaving the organization (Dogantekin et al., 2023). The relationship between coworker incivility and turnover intention is proven by this mediating effect of job satisfaction, which reduces job satisfaction and increases turnover probability.

Multiple studies have shown that there is a positive connection between rude behavior at work and employees' desire to quit (Namin et al., 2021) has revealed a strong positive link between these factors. The research shows a positive correlation between incivility and cynicism among co-workers, employees may consider leaving when they perceive incivility. Rahim and Cosby (2016) additionally suggest a model that is based on job performance, burnout, intent to leave, and workplace incivility as essential aspects for an organization to consider when an employee leaves. The research has found that the relationship between intention to leave and incivility is mediated by burnout. This means coworker incivility could lead to burnout which is another possible reason for leaving the organization. Therefore, we posit that:

*Hypothesis 2: Coworker incivility is positively related to turnover intention*.

#### 2.3.3 Emotional exhaustion and turnover intention

Emotional exhaustion has been widely recognized as a significant predictor of turnover intention. The Job Demands-Resources (JD-R) model posits that high job demands, such as workload and interpersonal conflicts, deplete employees' energy and lead to emotional exhaustion (Schaufeli and Bakker, 2004). This state of exhaustion diminishes employees' capacity to cope with job demands, thereby increasing their intention to leave the organization (Lee and Cho, 2020). Moreover, affective negativity—referring to long-lasting negative emotions such as irritability, frustration, and sadness—is associated with emotional exhaustion (Alessandri et al., 2020). The people who experience these negative feelings may think that their present jobs are emotionally exhausting. Some of them might become more determined to leave their jobs and start anew somewhere else in a quest to leave the negativity behind.

Also, emotional exhaustion can lead to a feeling of lack of control with regard to one's job and the belief that one is helpless (Gabriel et al., 2020). Employees could feel powerless to change their circumstances within the organization if they think their emotional wellbeing is constantly at risk due to their working environment. The desire to quit may grow in the desire to have a more controllable and emotionally less demanding work environment as a result of this lack of control. Moreover, emotional exhaustion and the decision to quit an organization can be affected by the general emotional atmosphere that prevails within it (Saleh et al., 2022). Employee emotional exhaustion is more likely to happen in an environment where there is high emotional demand, scarce support, or tolerance of uncivil behavior. Consequently, employees may feel more inclined to leave the job in search of a more emotionally balanced workspace. Peer interactions also influence emotional exhaustion and turnover intention (Prasad et al., 2023). The contagion effect may happen when the employees observe their co-workers announcing their plans to leave due to emotional exhaustion. This peer pressure can deepen their resolve to leave the organization which can lead to a chain effect within the workplace. Therefore, we posit that:

*Hypothesis 3: There is a positive relationship between emotional exhaustion and turnover intention*.

#### 2.3.4 Mediating role of emotional exhaustion

Numerous studies have provided evidence in support of the hypothesis that emotional exhaustion acts as a mediator in the beneficial link between coworker incivility and turnover intention. According to Shin and Hur (2019), incivility at work is prevalent and negatively affects employees. This incivility has the potential to cause emotional exhaustion, which increases turnover intention. Chami-Malaeb (2022) provided more evidence in support of this relationship by establishing that burnout, which includes emotional exhaustion, acts as a mediator in the relationship between workplace factors and nurses' intention to leave their jobs.

Emotional exhaustion plays a powerful mediating role, suggesting that employees who encounter incivility from their coworkers become emotionally depleted and eventually exhausted; their greater inclination to leave may then be affected. Wen et al. (2023) found that verbal abuse from coworkers had a higher influence on emotional exhaustion than external sources did, emphasizing the significance of incivility among coworkers in this relationship.

Demsky et al. (2014) found that psychological disengagement plays an indirect role in mediating work-family conflict caused by workplace aggression; they found this effect can be reduced through psychological detachment (the ability to mentally disengage from work during non-work time). Employees emotionally drained as a result of coworker incivility may find it more challenging to disengage from their jobs and take a step back, increasing turnover intentions and increasing turnover intentions.

“Social undermining” refers to any behavior which compromises society and is pertinent to this discussion as well. According to Eissa and Lester (2023), social undermining refers to actions that sabotage relationships, cause conflict, and impede the achievement of goals. Coworker incivility undermines relationships and fosters an unfavorable work environment, making it a form of social undermining. This social undermining could increase employees' turnover intentions after experiencing emotional exhaustion. Therefore, it is hypothesized that:

*Hypothesis 4: The positive relationship between coworker incivility and turnover intention is mediated by emotional exhaustion*.

#### 2.3.5 Moderating role of mindfulness

Extensive research has been done on the positive relationship between coworker incivility and turnover intentions, underscoring the negative impact of workplace incivility on employee retention (Namin et al., 2022). Nevertheless, recent studies have proved that mindfulness might act as a critical moderator, which is used by workers to perceive and respond to workplace incivility. Mindfulness is an important factor in this process, and it gives employees the skills of emotional regulation (Chen et al., 2022). When they face negative behavior from their colleagues, employees with more developed mindfulness tend to be better at controlling their emotions. They emphasize the importance of staying calm and not reacting in a rash manner, which makes it easier to cope with incivility (Chen and Hu, 2023). Thus, the employees who practice mindfulness tend to have low turnover intentions in reaction to emotional distress aggravation. Additionally, mindfulness is commonly known to be an effective stress-reduction tool, which makes it a beneficial resource for workers who are struggling with workplace stressors such as incivility among colleagues (Bartlett et al., 2021). Practicing mindfulness methods helps to build resilience against stress, therefore, reducing emotional exhaustion which is a common feature of incidents of incivility. Thus, the turnover intentions as a strategy for escaping stress are reduced.

Mindfulness in addition cultivates adaptive coping skills. Employees who develop mindfulness tend to respond to coworker incivility by engaging in problem-solving that is aligned with the organizational culture. Rather than terminating their job as a means of escaping, they join open discussions, negotiate, or get help from supervisors or human resources to deal with or correct uncivil behavior (Hawkes and Neale, 2020). Therefore, with these adaptive coping mechanisms that are used as an answer to incivility, the need for turnover is reduced. Also, mindfulness enhances interpersonal skills equipping employees with the skills they need to negotiate disagreements and incivility more effectively (Hawkes and Neale, 2020). Employees who are aware of their emotions are better equipped to manage difficult situations, talk to their co-workers about their concerns, and create a more harmonious and peaceful workplace. Mindfulness decreases the negative effects of coworker incivility on the intention to leave the job by encouraging positive interpersonal relationships.

Mindfulness serves as a buffer against the negative consequences of workplace uncivil behavior. It helps people to recover from negative events, thus reducing the emotional scars that can be the main reason for the intention to quit (Raza et al., 2018). The mindfulness of employees enables them to view incivility as a temporary challenge and therefore, focus on the present moment and not dwell on the past unfavorable events (Sauer et al., 2013). Shifting the focus instead permits employees to disconnect from the emotional consequences of past incivility episodes, which mitigates their influence on turnover intentions. Lastly, mindfulness has been linked with overall improved wellbeing (Huynh and Torquati, 2019). Individuals who practice this often tend to have a more positive outlook on life and work, thus perceiving their wellbeing as less dependent on external factors. This, in consequence, lowers their tendency to leave the organization because of the coworker's incivility. Therefore, it is posited that:

*Hypothesis 5: The positive relationship between coworker incivility and turnover intention is moderated by mindfulness*.

## 3 Methods

### 3.1 Sample and procedures

The participants in this study are nurses who work in North Cyprus public and private hospitals. The duration of data collection was 4 months from March to July 2023. The duration of data collection was 4 months from March to July 2023. In this study, we employed a cross-sectional research design, focusing on nurses working in 20 hospitals across North Cyprus. To determine the appropriate sample size, we used the rule of thumb formula proposed by Van Voorhis and Morgan (2007), which is calculated as n × 10 + 50, where n represents the number of variables. Given that our study included 18 variables, the required sample size was 230 nurses (18 × 10 + 50 = 230). The measurements used in the data collection were originally prepared in English language and then translated to Turkish language and back-translated into the English language to be sure doesn't lose the meaning (McGorry, 2000). To confirm the validity of the variable pilot study was conducted with 30 nurses who work in public and private hospitals in North Cyprus.

The study was purposive sampling, 300 questionnaires were distributed and 238 were collected back (79%). The majority of the respondents are female (71%) and worked in private hospitals (55.5%) with 29–39 years old (58.8%). Table 1 provides detailed information about the participants and the prevalence of coworker incivility and levels of turnover intention by gender, age, and organization (public/private). Our findings reveal no significant difference in coworker incivility experienced by male and female staff. However, men report higher levels of turnover intention. The youngest age group (29–39 years old) reports the highest levels of coworker incivility, while the oldest age group reports relatively lower levels of both coworker incivility and turnover intention. Additionally, private hospitals see more reports of coworker incivility and turnover intention compared to public hospitals.

**Table 1:** Information on the participant.

		**Coworker incivility**				**Turnover intention**			
	**N**	**Mean**	**SD**	***F***	***p***	**Mean**	**SD**	***F***	***p***
*Gender*				2.76	0.09			7.45	0.07
Male	69	3.91	0.90			3.61	1.12		
Female	169	3.64	1.20			3.12	1.28		
*Age*				15.11	0.00			11.21	0.00
18–28	19	2.67	1.27			2.96	1.46		
29–39	140	4.00	0.87			3.56	1.10		
40–50	73	3.59	1.23			2.94	1.29		
51and above	6	2.12	0.37			1.22	0.40		
*Organization*				17.80	0.00			102	0.00
Public	106	3.39	1.32			2.49	1.02		
Private	132	3.99	0.86			3.88	1.26		

N, SD, F, and P are the number of participants, standard deviation, F -test (difference of 2 variances test), and p-value (significant level) respectively.

### 3.2 Measurement

The survey consisted of two parts: the first part gathered demographic information, while the second part focused on coworker incivility, emotional exhaustion, mindfulness, and turnover intention. All construct variables were measured using a five-point Likert scale ranging from strongly disagree (1) to strongly agree (5).

#### 3.2.1 Co-worker incivility

Coworker incivility was assessed using four items adopted from Sliter et al. (2012), which originate from the Interpersonal Conflict at Work Scale developed by Spector and Jex (1998). The original scale has a Cronbach's alpha of 0.79, while the current study achieved a Cronbach's alpha of 0.94, indicating high reliability. Sample items include, “How often do coworkers ignore or exclude you while at work?” and “How often are coworkers rude to you at work?” Responses were recorded on a five-point Likert scale ranging from never (1) to very frequently (5).

#### 3.2.2 Emotional exhaustion

Emotional exhaustion was measured using the six-item Maslach Burnout Inventory for service occupations (Maslach and Jackson, 1981). Previous research has reported a Cronbach's alpha of 0.94 for this scale, confirming its high reliability (Anasori et al., 2020). In this study, the Cronbach's alpha was 0.92. Example items include, “I feel emotionally drained from my work,” and “I feel used up at the end of the workday”. Responses were recorded on a five-point Likert scale from never (1) to very frequently (5).

#### 3.2.3 Turnover intention

Turnover intention was assessed using three items based on the scale by Lum et al. (1998). Previous research has reported a Cronbach's alpha of 0.97, indicating high reliability (Yulianti, 2020). The Cronbach's alpha for this study was 0.87. An example item is, “In the last few months, have you ever thought seriously about looking for a nursing job at another hospital?” Responses were recorded on a five-point Likert scale from strongly disagree (1) to strongly agree (5).

#### 3.2.4 Mindfulness

To measure mindfulness as a state rather than a trait, the MAAS-State scale was used, which evaluates an individual's level of attention and awareness of their current environment. This scale consists of five items selected from the 15-item MAAS scale and reformulated to focus on the current situation rather than general characteristics (Brown and Ryan, 2003). All items were reverse-coded. The Cronbach's alpha for this study was 0.86. Sample items include, “I was doing something without paying attention,” and “I was finding it difficult to stay focused on what was happening.” Responses were recorded on a five-point Likert scale from never (1) to very frequently (5).

#### 3.2.5 Control variable

In this research, age was used as a control variable. Historically, turnover rates have been highest among young nurses. We assessed whether the control variable was significant in influencing the outcomes.

### 3.3 Data analyses

Data was analyzed using SPSS and Smart-PLS. The measurement model was evaluated for reliability using Cronbach's Alpha and assessed for convergent validity, discriminant validity, and confirmatory factor analysis. Mediation and moderation analyses were also conducted. The study's hypotheses were tested using structural equation modeling.

To prevent common method variance (CMV), three approaches were employed to reduce common method bias. First, the confidentiality and anonymity of the questionnaire were emphasized. Second, participants were encouraged to answer each question honestly, as there were no right or wrong answers. Third, all items were factor analyzed using Harman's single factor test (Podsakoff et al., 2003). CMV is considered problematic if a single factor explains more than 50% of the total variance. In our analysis, a single latent factor explained 45.07% of the variance, indicating that CMV is not a significant concern.

## 4 Result

The initial stage of the analysis involved assessing the measurement model (outer model) to ascertain the validity and reliability of the constructs under investigation. Subsequently, the significance of the parameters is examined within the inner model to substantiate the relationships between variables. Table 2 reports descriptive statistics for the key variables, such as mean, standard deviation, Cronbach's alpha coefficients, and correlations. Means scores demonstrate the average levels of each variable of the sample, while standard deviations indicate the variability of responses around the mean. Another example is that the participants in this study more or less had the same level of incivility from their co-workers, with only slightly differing responses, as measured by the mean of 3.72 and standard deviation of 1.12. Likewise, emotional exhaustion, turnover intention, and mindfulness had moderate to high mean scores, which identified a considerable divergence within the sample regarding the mentioned constructs. Internal consistency reliability is, therefore, satisfactory for all the important variables. As indicated by Cronbach's alpha coefficients that range from 0.859 to 0.941, the variables are stable across tests. These high alpha values indicate that the sum of the elements of each construct is reliably measured by the items representing the concept underlying the construct.

**Table 2:** Descriptive statistics of major variables.

	**Mean**	**SD**	**Cronbach's alpha**	**Composite reliability**	**AVE**	**1**	**2**	**3**
Co-worker Incivility	3.72	1.12	0.941	0.958	0.850			
Emotional Exhaustion	4.09	0.78	0.925	0.941	0.728	0.681^**^		
Turnover Intention	3.26	1.26	0.875	0.922	0.798	0.594^**^	0,516^**^	
Mindfulness	2.41	0.73	0.859	0.900	0.649	−0.657^**^	-0.709^**^	−0.507

SD and AVE are Standard Deviation and Average Variance Extracted. Mean, standard deviation, correlation result. ^**^denotes significance at 1 percent.

The correlation matrix describes the bivariate relationships between variables. These findings highlight that there is a positive relationship between co-worker incivility and emotional exhaustion (r = 0.681, *p* < 0.01) as well as co-worker incivility and turnover intention (r = 0.594, *p* < 0.01) which means that the higher the level of the employees' perceived incivility from their co-workers, the more they feel exhausted emotionally. Also, emotional exhaustion had a very strong positive relationship with turnover intention (r = 0.516, *p* < 0.01), indicating that the more emotional exhaustion one experienced, the greater the chances of leaving the job. However, there were significant negative correlations between mindfulness and co-worker uncivil behavior (r = −0.657, *p* < 0.01), emotional exhaustion (r = −0.709, *p* < 0.01), and turnover intention (r = −0.507, *p* < 0.01), implying that the higher the mindfulness, the lower the levels of perceived incivility, emotional exhaustion, and turnover intention.

### 4.1 Measurement of model evaluation (outer model)

Indicator loadings show the reliability of the items to their constructs and the variation explained by the construct. The acceptable loading level for convergent validity has been proposed as 0.708 (Hair et al., 2019). However, some researchers have suggested this threshold as 0.505 (Falk and Miller, 1992).

Internal consistency was evaluated using Cronbach's α, with results indicating acceptable reliability levels above 0.7 for all constructs, as recommended by Hair et al. (2006). Specifically, Cronbach's α values were 0.94 for coworker incivility, 0.87 for turnover intention, 0.92 for emotional exhaustion, and 0.85 for mindfulness. Construct validity was assessed through convergent validity, measured by the loading factor (λ) and average variance extracted (AVE). According to Fornell and Larcker (1981), the AVE should be at least 0.50, and the loading factor should exceed 0.70. As shown in Table 2, all constructs in this study met these criteria, with loading factors above 0.7 and AVE values >0.50, ensuring robust construct validity.

Discriminant validity checks the extent to which a variable differs from another variable. The parameter proposed by Fornell and Larcker (1981) compares the average variance extracted (AVE) with correlations between latent variables. A measurement model is said to have good discriminant validity if the correlation between latent variables is less than the square root of the AVE. The square root of the AVE is indicated by **bold** numbers on the diagonal of the correlation table (Table 3). Additionally, this study used the Heterotrait-Monotrait (HTMT) criterion suggested by Ali et al. (2018). The HTMT values, shown in **italics** in Table 3, were all less than one. Therefore, the scale used in this study has sufficient construct validity.

**Table 3:** Correlation between variables, square root of AVE and HTMT ratios.

	**Age**	**Coworker incivility**	**Emotional exhaustion**	**Mindfulness**	**Turnover intention**
Age	**1**				
Coworker incivility	0.055	**0.922**			
Emotional exhaustion	0.042	0.73	**0.853**		
Mindfulness	0.063	0.731	0.8	**0.806**	
Turnover intention	0.24	0.66	0.58	0.599	**0.893**

Unlike covariance-based structural equation modeling, PLS does not produce goodness-of-fit statistics. Instead, the analytical ability of the model is assessed using the R-square value. According to Falk and Miller (1992), an R-square value greater than 0.10 is acceptable. In our study, the R-square values exceeded this threshold. Additionally, Wetzels et al. (2009) suggest calculating the geometric mean of the average variance extracted (AVE) and R-square values as another measure of model fit. In our study, the AVE values were 0.85, 0.728, 0.798, and 0.649, while the R-square values were 0.47 and 0.58. The average AVE was 0.756, and the average R-square was 0.52. Multiplying these averages yielded 0.396, and taking the square root resulted in 0.62, indicating that our model has a medium fit according to Wetzels et al. (2009). Another criterion for goodness of fit is the Standardized Root Mean Square Residual (SRMR). In our study, the SRMR value was 0.068, which is within the acceptable range, supporting the conclusion that our model is a good fit.

### 4.2 Structural model testing (inner model)

The next step involves evaluating the structural model to test the relationships between coworker incivility, emotional exhaustion, mindfulness, and turnover intention. To determine the significance of these relationships, t-statistics and *p*-values were generated using Smart PLS 3.0 bootstrapping, as detailed in Table 4.

**Table 4:** Item loading to their variable.

**Factors**	**Indicators**	**Loadings**
Co-worker incivility	CWI 1. How often do coworkers ignore or exclude you while at work?	0.907
	CWI 2. How often do coworkers raise their voices at you while at work?	0.938
	CWI 3. How often are coworkers rude to you at work?	0.933
	CWI 4. How often do coworkers do demeaning things to you at work?	0.909
Emotional exhaustion	EE 1. I feel emotionally drained from my work	0.862
	EE 2. I feel used up at the end of the workday	0.857
	EE 3. I feel fatigued when I get up in the morning and have to face another day on the job	0.868
	EE 4. Working with people all day is really a strain for me	0.855
	EE 5.I feel burned out from my work	0.847
	EE 6.I feel frustrated by my job	0.830
Mindfulness	MIND 1. I was finding it difficult to stay focused on what was happening.	0.906
	MIND 2. I was doing something without paying attention.	0.929
	MIND 3. I was preoccupied with the future or the past	0.871
	MIND 4. I was doing something automatically, without being aware of what I was doing	0.628
	MIND 5. I was rushing through something without being really attentive to it	0.639
Turnover intention	TI 1. In the last few months have you ever thought seriously about looking for a nursing job at another hospital?	0.906
	TI 2. In the last few months have you ever thought seriously about looking for a non-nursing job?	0.864
	TI 3. Taking everything into consideration, how likely is it that you will make a serious effort to and a new job within the next year?	0.901
Model fit statistic	SRMR = 0.068	

#### 4.2.1 Direct effect testing

Hypothesis 1: This hypothesis proposes that coworker incivility has a significant positive relationship with emotional exhaustion. The results support this hypothesis, showing a strong positive effect (β = 0.686, *p* < 0.001), thus confirming H1.

Hypothesis 2: This hypothesis suggests that coworker incivility significantly and positively relates to turnover intention. The findings support this hypothesis as well (β = 0.405, *p* < 0.001), thus confirming H2.

Hypothesis 3: This hypothesis proposes that emotional exhaustion has a significant positive relationship with turnover intention. The results show that this relationship is significant (β = 0.165, *p* < 0.05), thus supporting H3.

#### 4.2.2 Mediating effect testing

Hypothesis 4 proposes that Emotional Exhaustion has a mediation role between co-worker incivility and turnover intention and the result shows that these relations are significant and H4 accepted (β = 0.113, *p* < 0.05).

#### 4.2.3 Moderating effect testing

Hypothesis 5 proposes that mindfulness moderates the relationship between coworker incivility and turnover intention. Specifically, when mindfulness levels are high, the effect of coworker incivility on turnover intention becomes significant and negative. The results confirm this hypothesis (β = −0.124, *p* < 0.05), indicating a significant moderating effect of mindfulness.

**Figure 2:**
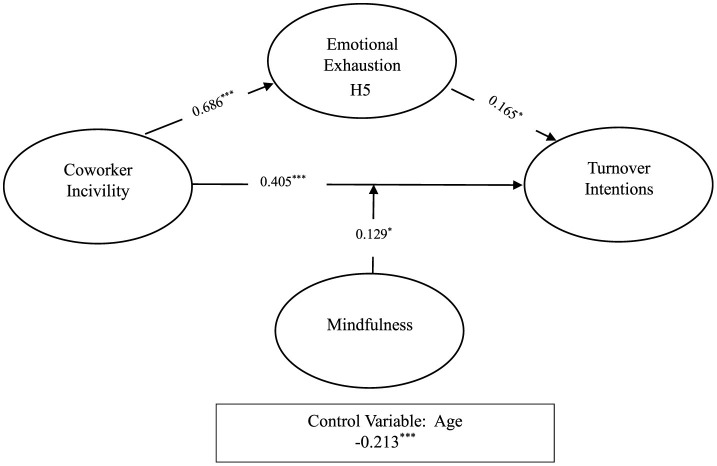
Results of the structural model. *P*-values *, **, and *** are statistically significant at 5 and 1 percent respectively.

Figure 2 illustrates this moderation effect. The graph shows that with high levels of mindfulness, turnover intention decreases even in the presence of coworker incivility. Conversely, when mindfulness levels are low, turnover intention increases under the same conditions. This demonstrates the buffering role of mindfulness in reducing the negative impact of coworker incivility on turnover intention.

**Figure 3:**
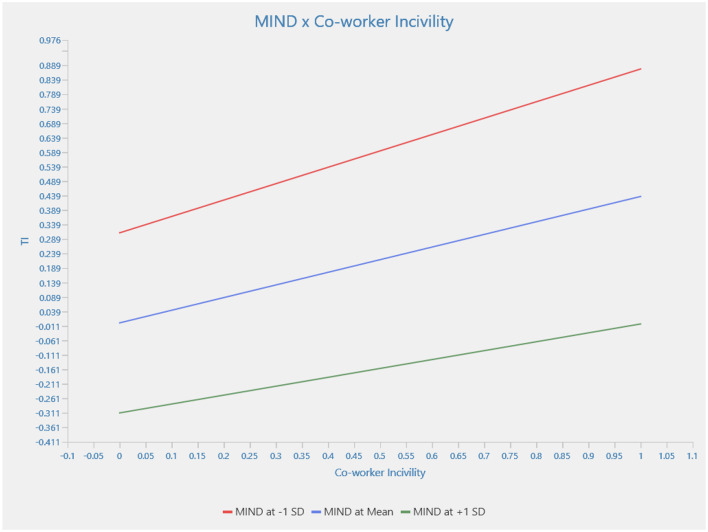
The interaction effect between mindfulness and co-worker incivility on employee turnover intention.

Figure 3 presents a detailed overview of the Structural Equation Model results. All hypotheses were found to be significant and acceptable.

## 5 Discussion

This study examined the relationship between coworker incivility and turnover intention among nurses working in public and private hospitals. The findings of the study revealed that the incivility that nurses perceived from their coworkers increased their turnover intention.

The study model developed in this research draws upon the Job Demand-Resources theory in the context of North Cyprus. This study aims to contribute to the existing literature by examining the relationship between co-worker incivility and turnover intention within healthcare organizations in North Cyprus, with emotional exhaustion serving as a mediator and mindfulness as a moderator.

Our analysis revealed that all hypotheses were supported by the data. From Table 5 we can see all the detailed information. Based on this we have main findings. Firstly, we found a positive relationship between co-worker incivility and emotional exhaustion (*p* < 0.001). Results agreed with the findings of previous studies by Parray et al. (2023) and Butt and Yazdani (2021). Workplace incivility involving behaviors such as rudeness and disrespect toward coworkers creates a stressful workplace environment, which in turn results in emotional exhaustion among employees. This finding is in line with the research conducted by Parray et al. (2023), which showed that the employees who were exposed to the incivility of their colleagues experienced more emotional exhaustion. For instance, Butt and Yazdani (2021) argued that the civil behaviors of coworkers make employees to be emotionally exhausted, which is in line with the findings of the present study.

**Table 5:** Summary of direct and indirect effects.

	**Beta**	**SD**	**T static**	***P* value**	**Decision**
1.Co-worker incivility → emotional exhaustion	0,686	0,042	16,95	0,000	Accepted
2.Co-worker incivility → turnover intention	0,405	0,067	6,094	0,000	Accepted
3.Emotional exhaustion → turnover intention	0,165	0,079	2,085	0,037	Accepted
4.Mindfulness → turnover intention	-0,342	0,102	3,361	0,001	Accepted
**Mediation**					
5. Co-worker incivility → emotional exhaustion → turnover intention	0,113	0,055	2,062	0,039	Accepted
**Moderation**					
6. Mindfulness^*^co-worker incivility → turnover intention	-0,129	0,053	2,449	0,015	Accepted

This relationship suggests that increased experiences of incivility among nursing staff are associated with higher levels of emotional exhaustion, a key component of burnout. In the nursing profession, co-worker incivility can take many forms, such as rude or dismissive comments, exclusion from work-related activities, and non-verbal hostility. These negative interactions can disrupt teamwork, decrease job satisfaction, and impair the overall work environment (Ostroff et al., 2023). When nurses experience incivility, it undermines their emotional well-being, leading to increased stress and emotional exhaustion. Emotional exhaustion, in turn, affects their capacity to provide high-quality patient care, which can result in adverse patient outcomes and increased turnover rates (Wu et al., 2024). Several mechanisms explain why co-worker incivility leads to emotional exhaustion in nurses. First, incivility consumes psychological resources, as nurses must expend additional emotional energy to cope with negative interactions (Woo and Kim, 2020). Second, incivility can lead to a hostile work environment, reducing the sense of community and support among staff, which is crucial for managing the emotional demands of nursing (Heaslip and Crossthwaite, 2022). Third, persistent exposure to incivility can erode nurses' professional identity and job satisfaction, further contributing to emotional exhaustion (Ostroff et al., 2023).

In addition, the results of our study revealed a highly statistically significant positive relationship between co-worker incivility and turnover intention (*p* < 0.001), thus supporting the second hypothesis. This result is consistent with the previous studies (and Hendryadi and Zannati, 2018; Tricahyadinata et al., 2020; Namin et al., 2021; Aman-Ullah et al., 2023). Aman-Ullah et al. (2023) determined that coworker incivility is a reliable predictor of employees' turnover intentions in healthcare organizations, supporting our findings. Tricahyadinata et al. (2020) also found a direct connection between co-worker incivility and nurses' intention to resign, which was consistent with our study. The relationship between emotional exhaustion and the intention to quit is also positive, which was confirmed by the results of the study by Butt and Yazdani (2021), Parray et al. (2023), and Rajendran et al. (2020). So, employees who are emotionally exhausted at high levels are more likely to think of quitting their jobs, as they see the current job as exhausting and having no meaning. Butt and Yazdani (2021) addressed emotional exhaustion as a significant predictor of healthcare professionals' turnover intention, which is in line with our research findings. The relationship between co-worker incivility and turnover intention is underpinned by several psychological and organizational factors. Incivility negatively affects job satisfaction and organizational commitment by creating a hostile work environment. Nurses subjected to frequent incivility may feel undervalued and unsupported, leading to a diminished sense of belonging and a lower attachment to the organization (Alsadaan et al., 2024). Consequently, this sense of disconnection can increase turnover intention as nurses seek more supportive and respectful work environments. Further, the negative impact of incivility on emotional resources and mental health can exacerbate turnover intention. Persistent exposure to incivility can lead to burnout, characterized by emotional exhaustion, depersonalization, and a reduced sense of personal accomplishment (Parray et al., 2023). As burnout intensifies, nurses may contemplate leaving their jobs as a means of escaping the detrimental work conditions, thus elevating turnover intention.

The study also investigates the mediation role of emotional exhaustion. Emotional exhaustion mediated the relationship between co-worker incivility and turnover intention (*p* < 0.005). Emotional exhaustion functions as a critical mediator in this relationship by amplifying the impact of co-worker incivility on turnover intention. Research indicates that emotional exhaustion directly affects turnover intention by diminishing job satisfaction and reducing organizational commitment (Alsadaan et al., 2024). Nurses who experience high levels of emotional exhaustion may perceive their work environment as increasingly unsupportive and hostile, reinforcing their intent to leave (Pang et al., 2020). Consequently, the negative effects of co-worker incivility are magnified through this emotional exhaustion, making it a pivotal factor in explaining why employees are more likely to contemplate turnover. This mediation effect has been demonstrated in previous studies by Moon and Morais (2022) and López-Cabarcos et al. (2021), showing that emotional exhaustion is a key mechanism through which worker incivility causes work turnover intention. Moon and Morais (2022) showed that the relationship between co-worker incivility and turnover intention among healthcare employees was significantly mediated by emotional exhaustion, which is highly consistent with our findings.

Lastly, our result was in favor of the moderating role of mindfulness that influences the relationship between co-worker incivility and turnover intention, supporting our fourth hypothesis (*p* < 0.005). Mindfulness, which is the ability to be fully aware and accepting one's thoughts and feelings in the present moment, was proved to be the factor that reduces the negative impact of co-worker incivility on turnover intention. Mindfulness can moderate the relationship between co-worker incivility and turnover intention by influencing how employees perceive and respond to incivility. Mindful individuals are better equipped to manage their emotional responses and maintain a balanced perspective, even in the face of negative interactions (Parray et al., 2023). This heightened emotional regulation allows them to experience less distress and burnout from incivility, thereby mitigating its impact on their intention to leave their jobs. Research has shown that mindfulness is associated with reduced stress and improved emotional resilience, which can act as a buffer against the adverse effects of workplace incivility (Hülsheger et al., 2021). By fostering a greater awareness of their internal states and a more objective view of external stressors, mindful employees may be less likely to experience the emotional exhaustion that typically drives turnover intention (He et al., 2023). Consequently, they are better able to cope with co-worker incivility without letting it unduly influence their decision to stay or leave their position. It is in line with the results of Yu et al. (2020) and Yang and Xu (2023), which indicate that mindful individuals tend to be better at coping with workplace challenges and have lower turnover intentions. As Yu et al. (2020) have shown, mindfulness serves as a moderator between mistreatment at the workplace and turnover intention, thus, substantiating our finding.

From our research, we show that there is a positive relationship between coworker incivility and turnover intention in the nurses who participated in this study. Our findings suggest that a suitable working environment should be provided to increase the mindfulness of healthcare sector employees. In this study, one of the biggest inhibitors of mindfulness was reported to be workplace incivility. When employees have a low level of mindfulness, this leads to emotional exhaustion and ultimately to an environment of insecurity and fear that limits innovation and creativity (Said and Tanova, 2021). While recent literature has demonstrated the importance of mindfulness, most studies have treated it as a trait and conceptualized it as a determinant or boundary condition. The current study, however, examines the role of mindfulness not as a fixed trait but as a state that is influenced by workplace events such as incivility.

## 6 Conclusion

This study investigates the relationship between coworker incivility and turnover intention among nurses in both public and private hospitals in North Cyprus. The findings reveal a significant positive correlation: nurses experiencing incivility from coworkers are more likely to consider leaving their jobs. This relationship is mediated by emotional exhaustion, meaning that incivility leads to greater emotional fatigue, which in turn increases turnover intention. Additionally, mindfulness serves as a moderator, with higher levels of mindfulness reducing the impact of incivility on turnover intentions.

Employee turnover is a pressing issue across various industries, particularly in healthcare, which suffers from some of the highest turnover rates among doctors and nurses. Workplace incivility, a form of negative behavior, is linked to numerous mental and physical health problems for nurses. Studies highlight the substantial costs associated with employee turnover, including the burden of recruiting and onboarding new, often inexperienced staff, which places additional responsibilities on current employees.

In North Cyprus, nurses in healthcare institutions report dissatisfaction due to incivility from colleagues, which exacerbates emotional fatigue and increases the likelihood of contemplating turnover. In public hospitals, nurses may seek transfers to other departments to escape such environments, whereas those in private hospitals might opt to leave their jobs entirely. However, nurses with higher mindfulness levels are less affected by coworker incivility regarding their turnover intentions, suggesting that mindfulness can buffer the negative effects of incivility.

This study contributes to the existing body of knowledge by applying the Job Demands-Resources theory to the healthcare sector in North Cyprus, marking the first investigation of its kind in this context. The insights gained enrich our understanding of the dynamics within healthcare organizations and offer practical implications for reducing turnover through mindfulness interventions and addressing workplace incivility.

### 6.1 Theoretical implications

This study contributes significantly to the theoretical framework of workplace incivility, emotional exhaustion, mindfulness, and turnover intention, particularly within the context of the nursing profession in North Cyprus. The utilization of the Job Demands-Resources (JD-R) model provides a robust theoretical foundation for understanding these complex relationships. This research advances the JD-R model by integrating mindfulness as a job resource, thereby offering a nuanced perspective on how mindfulness can mitigate the adverse effects of job demands, specifically coworker incivility, on emotional exhaustion and turnover intention. This integration extends the JD-R model, providing empirical evidence on the effectiveness of mindfulness in reducing emotional strain and improving employee retention in high-stress environments like healthcare.

Moreover, this study addresses a significant gap in the literature by focusing on the under-researched context of North Cyprus. By examining the cultural, social, and organizational factors unique to this region, the research offers valuable insights into how these contextual elements influence workplace behaviors and outcomes. This contextualization is critical for developing a comprehensive understanding of coworker incivility and its consequences, thus enriching the existing body of knowledge on workplace incivility in different cultural settings.

The findings also highlight the importance of considering cultural norms and values in shaping workplace interactions. The study's focus on North Cyprus, a region evolving into a multicultural society, underscores the need for organizations to adapt their cultural values and practices to prevent workplace incivility and promote a respectful work environment.

### 6.2 Practical implications

The practical implications of this study are profound, particularly for healthcare organizations aiming to improve workplace environments, enhance employee wellbeing, and reduce turnover rates. The identification of mindfulness as a critical job resource offers practical strategies for organizations to implement mindfulness training programs. These programs can equip employees with skills to manage emotional stress and respond more effectively to incivility, thereby reducing emotional exhaustion and the intention to leave the organization.

Healthcare managers can utilize the findings to develop targeted interventions aimed at fostering a supportive and respectful work environment. By addressing the root causes of coworker incivility and promoting a culture of mindfulness, organizations can mitigate the negative impacts of incivility on employee wellbeing and retention. This is particularly important in the healthcare sector, where high turnover rates can severely impact patient care and organizational performance.

Additionally, the study's insights into the unique challenges faced by nurses in North Cyprus can inform the development of culturally sensitive policies and practices. Understanding the specific cultural, social, and organizational dynamics that contribute to workplace incivility in this region allows for the creation of tailored strategies to address these issues. For example, training programs that emphasize respect for cultural diversity and effective communication can help reduce instances of incivility and improve overall workplace harmony.

By integrating these practical recommendations, healthcare organizations can not only enhance the wellbeing and job satisfaction of their employees but also improve organizational outcomes, including patient safety and care quality. The study's findings provide a compelling case for the adoption of mindfulness practices and culturally informed interventions as effective tools for managing workplace incivility and its associated challenges.

### 6.3 Limitations and future directions

While this study offers valuable insights into the relationships between coworker incivility, emotional exhaustion, mindfulness, and turnover intention, certain limitations should be acknowledged that does not undermine the overall methodology. The focus on nurses in North Cyprus may limit the applicability of the findings to other regions and professions. Expanding future research to include a more diverse sample would enhance the generalizability of the results. Additionally, the cultural and organizational context of North Cyprus, with its unique characteristics, may influence the study's outcomes. While these findings are valuable, examining similar phenomena in different cultural and organizational settings would provide a broader understanding.

Building on the strengths of the current study, several avenues for future research can be recommended. Examining the relationships between coworker incivility, emotional exhaustion, mindfulness, and turnover intention in various cultural contexts through cross-cultural comparisons can help identify universal and culture-specific dynamics of workplace incivility. Investigating these constructs in other high-stress professions, such as teaching, law enforcement, or emergency services, can provide comparative insights and enhance the generalizability of the findings.

Implementing longitudinal studies would enable researchers to track changes over time, offering deeper insights into the causal relationships and long-term effects of workplace incivility and mindfulness interventions. Employing a combination of qualitative and quantitative methods could provide a more comprehensive understanding. Interviews and focus groups, for example, can offer nuanced insights that quantitative data alone may not capture.

Future research should also explore the effectiveness of specific mindfulness-based interventions and other organizational strategies aimed at reducing workplace incivility. Experimental and quasi-experimental designs can assess the impact of these interventions on employee well-being and turnover intentions. By addressing these limitations and pursuing these research directions, future studies can build on the robust methodology of the current study and further contribute to the understanding and mitigation of workplace incivility in various contexts.

## Data availability statement

The raw data supporting the conclusions of this article will be made available by the authors, without undue reservation.

## Ethics statement

The studies involving humans were approved by Eastern Mediterranean University Academic Evaluation Committee Research Advisory Board Research and Publication Ethics Board. The studies were conducted in accordance with the local legislation and institutional requirements. The participants provided their written informed consent to participate in this study.

## Author contributions

GH: Conceptualization, Data curation, Formal analysis, Investigation, Methodology, Software, Writing – original draft, Writing – review & editing. Mİ: Supervision, Writing – original draft, Writing – review & editing.
